# GNSS spoofing detection using a maximum likelihood-based sliding window method

**DOI:** 10.1371/journal.pone.0237146

**Published:** 2020-08-28

**Authors:** Seongkyun Jeong

**Affiliations:** Department of Human Intelligence Robot Engineering, Sangmyung University, Cheonan, Republic of Korea; University Campus Bio-Medico of Rome, ITALY

## Abstract

The Global Navigation Satellite System is vulnerable to interference signals that can potentially disable the system, because the signal strength tends to be very weak. Interference such as jamming, which disables the receiver via excessively high signal strength in the satellite navigation frequency band, and spoofing, which induces the receiver to output erroneous position and time data via signals similar to actual navigation signals, disrupt satellite navigation systems. As the threat of interference is increasing, considerable research effort has been expended in an attempt to deal with it in various ways. Spoofing attacks are especially difficult to detect. This paper deals with a technique to detect a spoofing signal and to mitigate attacks on satellite navigation systems. The satellite navigation signal is influenced by the navigation satellite itself and errors due to environmental factors, and spoofing signal detection should be well reflected in the navigation signal. Especially, in the case of mobile receivers, it is not easy to detect a spoofing signal because the exact position of the receiver cannot be known. To detect a spoofing signal, additional hardware may be required; in some cases, heterogeneous sensors, such as inertial sensors, may be used. The technique introduced in this paper effectively discriminates spoofing signals based only on receiver measurements, without the need for additional devices. It generates test statistics based on the pseudorange, which is the measured value of the receiver position, and detects spoofing signals by setting the monitoring interval according to a “sliding window”. Because the proposed method uses output data and measurements obtained from the receiver, it can be applied to general receivers at low cost.

## Introduction

Satellite navigation signals are vulnerable to interference signals; the ability to detect such interference is necessary, especially in systems in which high reliability is of the utmost importance. Satellite navigation interference can range from jamming, which disables the receiver via excessively high signal intensity in the satellite navigation frequency band, and spoofing, which induces the receiver to output erroneous position and time data via signals similar to actual navigation signals, thereby disrupting satellite navigation systems. Because a navigation receiver continuously generates position and time information, even if it is disrupted by a spoofing signal, users may not immediately notice navigation signal abnormalities. Erroneous position/time information could affect military and economic applications, for example, resulting in extensive damage or losses. Better techniques for detecting spoofing signals are required to prepare for ongoing and sophisticated spoofing attacks made on a system, particularly those in which high reliability is a necessity (e.g., those providing navigation information for aircraft). The detection methods can be divided into three types, based on the use of: (i) the signal processing module of the receiver [1], (ii) the data of the receiver output [2], and (iii) another sensor, such as an inertial sensor [3, 4]. The first type requires changing the signal processing module; additional antennas may be required depending on the exact method employed [5, 6]. The third type requires an extra sensor in addition to the Global Navigation Satellite System (GNSS) receiver. The second method which uses the receiver output can detect spoofing with low-cost because it does not need additional equipment and can be applied in existing receiver. However, it is not easy to accurately detect the spoofing signal by analyzing the output of a receiver.

There are various methods to detect spoofing signals based on the output of the receiver, such as signal strength test [7], measurement test [8, 9], navigation message test, and navigation solution test. Since spoofing factors are diverse and can be monitored from various measurements, the outputs of receivers should be checked by multiple methods to accurately determine whether or not spoofing occurs actually.

Recently, GNSS augmentation system can be applied when we use a satellite navigation system to improve navigation performance and integrity. The spoofing detection method by examining a navigation solution using GNSS augmentation system has also been proposed [10]. This method can comprehensively analyze the effects of spoofing signals in terms of checking the rationality of the navigation solution of the receiver. This method could be widely used when correction information is commonly used in the future.

However, for checking of the rationality of navigation solution, it is difficult to maintain detection performance continuously in all operating areas of a receiver, because GNSS continuously has error elements and the position of a receiver changes in a moving receiver. The reason is that receiver of the satellite navigation generates errors and drifts in the navigation solution according to the error environment and the position of the navigation satellite. Therefore, in order to detect spoofing, continuous reset is required according to changing environment.

In this paper, we proposed a method that can continuously detect spoofing even if errors or drifts occur in navigation solution. This method detects spoofing through rationality test of navigation solutions using GNSS augmentation system. We also compared existing available techniques for deciding final spoofing attack.

The proposed method examines the correlations of measurement values with navigation solutions to identify spoofing signals according to changes in navigation solutions. To determine rationality, it may be possible to perform a rationality test on individual or accumulated measurement epochs. For rationality tests performed on individual measurement epochs, commonly used analysis methods include receiver autonomous integrity monitoring (RAIM) and the chi-square test. The RAIM method can be used as a general monitoring technique to detect signal abnormalities, but it is only possible to detect a spoofing signal when the correlations between the measured values and navigation solutions are disrupted in the measurement epoch, which may occur if several channels are spoofed, for example [11]. Using the chi-square test, a spoofing signal can be detected successfully only when a significant difference occurs with respect to the authorized signal. To avoid these shortcomings, a method that accumulates measured values can be used; for example, the cumulative sum control chart (CUSUM) method may be applied [12].

The CUSUM method detects signal anomalies after a certain period of time, even when only a small signal perturbation occurs during the measurement time frame. This method can be used to detect continuous low-level abnormalities, including spoofing signals. The GNSS signal has the characteristic that the steady state changes continuously with changes in the satellite signal or the position of the receiver. In this paper, we propose a monitoring technique that addresses the problems of existing methods and can be applied to receivers at low cost. The detection process is focused as point of practical application, rather than consideration of test statistics. The proposed maximum likelihood-based “sliding window” method can detect spoofing signals by comparing signal characteristics before and after spoofing over a certain period. This method enhances spoofing signal detection performance. Therefore, the purposes of our study were to review the limitations of existing methods and to introduce a new spoofing detection technique that reflects the characteristics of the navigation signal.

In this paper, we will discuss the detection of spoofing signals by examining the correlation between satellite navigation measurement values and navigation solutions in an effort to detect spoofing attacks on satellite navigation systems.

### Review of disadvantages in conventional detection techniques

For checking of the rationality of the navigation solution in the satellite navigation system, there is a RAIM method that checks the rationality of the navigation solution by comparing the predicted pseudorange through a system matrix and the actual measured pseudorange. When a spoofing signal is applied and if the rationality between measurement and navigation solution is maintained, the abnormality of the navigation solution cannot be detected in this method. In case of a receiver using GNSS augmentation system, which is the object of detection in this paper, the pseudorange is estimated based on the navigation solution applying the correction information instead of the predicted value through the system metrics [11] and the pseudorange measured by the receiver.

For comparison of pseudoranges, the chi-square test can be applied. However, this method has a limitation in that it is difficult to distinguish between spoofing signal and measurement vibration when an instantaneous error occurs. The cumulative technique can be used to compensate for the instantaneous error. The CUSUM technique, which is a typical cumulative technique, can be applied. However, if drift of the navigation solution continuously occurs, detection is difficult due to the increase of error index even in normal operation. Each method has representativeness of detecting system error. This chapter introduces a method to check the rationality of navigation using representative techniques and describes limitations in detecting spoofing.

Conventional techniques for detecting anomalies in navigation receivers according to measured values are based on RAIM, where the optimal receiver position is determined using the pseudoranges measured for each satellite. The RAIM method checks whether the correlation between the calculated navigation solution and individual measurements is appropriate. Eqs (1) and (2) describe the RAIM method for detection of anomalies in measured values [11]:

$$V=(A(A^{T}\Sigma^{-1}A{)}^{-1}A^{T}\Sigma^{-1}-I)\varepsilon ,$$ (1)

$$test=\sqrt{V^{T}\Sigma^{-1}V}.$$ (2)

Here, **A** is a design matrix, **Σ** is a covariance matrix, and **I** is an identity matrix. **ε** represents the difference between the measured pseudorange and the estimated pseudorange. The test result for each measurement is the chi-square distribution in the steady state; the degree of freedom (*q* = *n* − 4) of the chi-square distribution is expressed by the number of satellites (*n*).

The RAIM method is capable of detecting a spoofing signal introduced to several channels. In this case, the correlation between the measurement solution and the navigation solution will not match. However, in cases of retransmission only, or when a spoofing signal is applied to all channels, a correlation between the measured values and navigation solution is established, such that it is impossible to detect the spoofing signal [13].

There is a method available for monitoring changes in the measurement values, as opposed to checking the correlation between the navigation solution and the measurement values. This method monitors the discrepancy between the actual and calculated measurement values based on the satellite and receiver positions; it determines the spoofing signal when this value changes and thus can compensate for the limitations of the RAIM technique.

Inspection of changes in measurement values is important during continuously monitoring of the difference between the measured and estimated pseudoranges. By monitoring the difference in the pseudoranges, it is possible to detect a spoofing attack using only a rebroadcasting device. The satellite position is determined by detecting changes in time error of the satellite navigation receiver. Since this is not a simple rationality check, there is a bias term in accordance with each satellite position and error element. To reduce the bias term and improve performance, the correction information derived from the augmentation system can be applied. Eq (4) shows the calculation used to obtain the difference between the corrected and estimated pseudoranges. Eq (5) shows the meaning of the pseudorange difference [10].

$$\rho_{e}^{i}=X^{i}-X_{u},$$ (3)

$$e^{i}=\rho_{c}^{i}-\rho_{e}^{i},$$ (4)

$$\rho_{c}^{i}-\rho_{e}^{i}=\rho^{i}+CR^{i}-\rho_{e}^{i}=\|X^{i}-X\|+b+{\tilde{\varepsilon }}_{\rho }^{i}+CR^{i}-\|X^{i}-X_{u}\|.$$ (5)

Here, $\rho_{e}^{i}$ is the estimated pseudorange of the *i*-th satellite, *X*^*i*^ is the *i*-th satellite position, *X*_*u*_ is the calculated receiver position, *e*^*i*^ is the pseudorange difference of the *i*-th satellite, $\rho_{c}^{i}$ is the corrected pseudorange of the *i*-th satellite, *ρ*^*i*^ is the measured pseudorange of the *i*-th satellite, *CR*^*i*^ is the correction value of *i*-th satellite, *X* is the real position of the receiver, *b* is the receiver bias, and ${\tilde{\varepsilon }}_{\rho }^{i}$ is the error element of the *i*-th satellite. The difference between the pseudoranges can be estimated by correcting the error in the correction information and maintaining a constant value if the calculated satellite position is close to the actual position in the steady state, as described by Eq (5).

The chi-square test detects signal abnormalities by comparing the deviation in the test statistics from the existing average value [14]:

$$test={(E-M_{G})}^{T}\Sigma^{-1}(E-M_{G}),$$ (6)

$$E={\left[e^{1}e^{2}\cdots e^{n}\right]}^{T},\;M_{G}={\left[\mu_{G}^{1}\mu_{G}^{2}\cdots \mu_{G}^{n}\right]}^{T}.$$ (7)

Here, **E** is a vector of the pseudorange difference (*e*) and **M**_**G**_ is a vector of the mean values (*μ*_*G*_). In this method, the degree of deviation from the average value is examined. Due to the properties of the navigation signal, which has various error factors, it is highly probable that a false alarm will be initiated by temporary measurement error. In particular, in the navigation signal, the error component may temporarily increase due to environmental factors. Therefore, continuous monitoring is needed to detect the spoofing signal.

CUSUM can be introduced for continuous monitoring. Monitoring the average change in the cumulative sum can be used for determining the abnormality of signals when the test statistic is continuously outside of the average range [15].

$$G_{0}=0,$$ (8)

$$G_{i}=\text{max}(0,\;G_{i-1}+(E_{i}-M_{G})\Sigma^{-1}(E_{i}-M_{G})-k),$$ (9)

$$k=m+{\left(M_{B}-M_{G}\right)}^{T}\Sigma^{-1}(M_{B}-M_{G}),$$ (10)

$$M_{B}={\left[\mu_{B}^{1}\;\mu_{B}^{2}\;\ldots \;\mu_{B}^{m}\right]}^{T}.$$ (11)

Here, *G* is the cumulative sum, **M**_*B*_ is a vector of abnormal state mean values (*μ*_*B*_), and *k* is a reference value. CUSUM detects the spoofing signal when the value of *G* exceeds the threshold value. This technique is effective for detecting spoofing signals, even faint ones, via accumulation of signal data. If a receiver is affected by a spoofing signal, the cumulative sum technique is effective, as changes in the average value occur continuously when tracking a spoofing signal.

**Fig 1:**
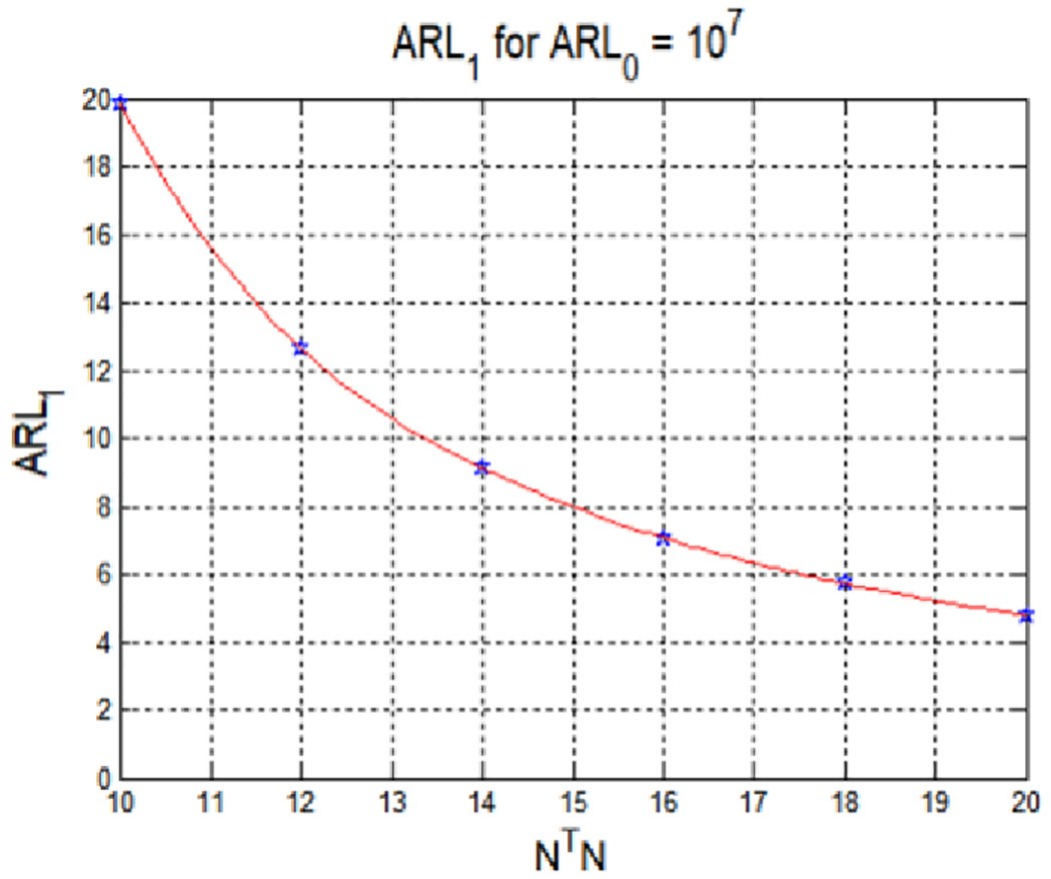
Threshold for spoofing signal detection according to the average run length.

The performance of the CUSUM method is expressed in terms of the average run length (ARL), as opposed to the probability of a false alarm or signal detection failure. The in-control ARL is the ARL required to detect an error in the normal state; the value is opposite to the probability of a false alarm For example, if the probability of a false alarm is 10^−7^, the corresponding value of the in-control ARL is 10^7^ [16]. If the required in-control ARL and abnormal state **M**_*B*_ are set, the threshold value can be determined using Eq (9) and numerical analysis [17]. Fig 1 shows the threshold value according to the abnormal state **M**_*B*_ obtained via the in-control ARL, in which Δ is given by

$$\Delta ={\left(M_{B}-M_{G}\right)}^{T}\Sigma^{-1}(M_{B}-M_{G}).$$ (12)

To apply this technique, an analysis of steady state average values must be performed. In the case of the navigation signal, drift is also present in the measured value because the satellite position changes continuously; additionally, the error due to environmental factors (e.g., the ionosphere) changes. Thus, the measurement value is affected by the geometric position of the satellite. To apply the CUSUM technique, the average value must be corrected continuously for comparison with the results of the RAIM method. Although both the chi-square test and CUSUM technique are effective for detecting changes in the spoofing signal, unlike the RAIM technique, the mean and standard deviation, which are the discrimination criteria for the spoofing signal, must be predicted correctly. It is impossible to maintain constant average test statistic values for the navigation signal when the position and altitude of the satellite are constantly changing. Also, because the receiver position changes in real time, certain restrictions must be applied during operation of the receiver. Various types of spoofing signals may be encountered; although the abovementioned methods can detect many spoofing signals, a more robust and practical method is needed.

### Spoofing detection algorithm using a maximum likelihood-based sliding window method

In this study, we introduce a technique to detect signal anomalies by comparison of the signal before and after spoofing using an “inspection window” in real time; this approach overcomes the disadvantages of existing detection techniques. The proposed method collects measured values over a certain period of time, sets a normal criterion, and then checks the input of the spoofing signal against the inspection window. By examining differences in the signal before and after the spoofing event, the spoofing signal input should be determined. If the difference between the measured and estimated pseudoranges is monitored continuously, any signal anomalies (relative to the steady state) induced by the spoofing signal can be clearly seen. The pseudorange is estimated based on the calculated receiver and satellite positions. Navigation signal abnormalities can be detected easily for a fixed receiver by comparing the calculated and reference positions. However, because it is difficult to apply this method to a mobile receiver, the difference in pseudorange is generally checked instead. In the case of the RAIM technique, only the correlation between the pseudoranges is examined. In this paper, differences in the pseudoranges are monitored continuously over time.

**Fig 2:**
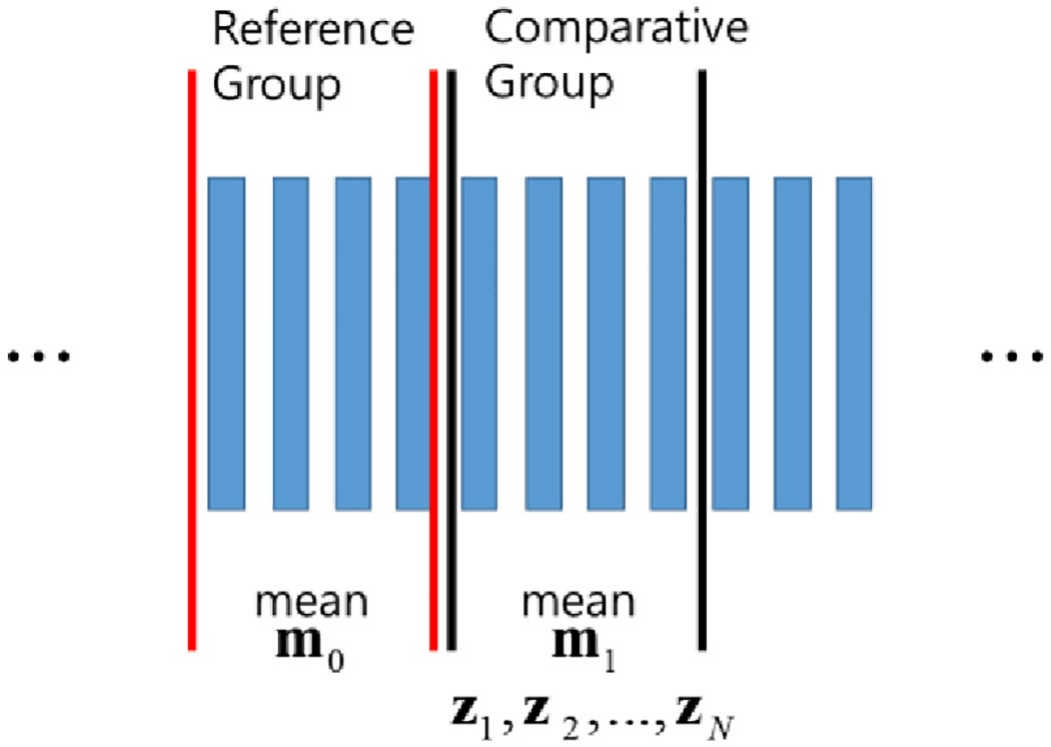
“Sliding window” concept.

Regarding the mobile receiver, it is not possible to easily and accurately identify the actual satellite position and thus detect a spoofing attack. It is also difficult to determine normal and abnormal signal states in advance. To solve this problem, we assume a normal satellite navigation signal during tracking. The measurement values collected over time are divided into reference window (reference group) and comparative window (comparative group) values. In the steady state, the reference window and comparative window values are similar. If the comparative window has abnormal measurement values, it suggests a spoofing signal. This method is referred to as the sliding window method, where the measured values shifts over time while the reference and comparison windows remain fixed. Fig 2 shows the concept of dividing measured values into reference and comparative window values using the sliding window approach. Assuming that N samples are obtained from the reference and comparative windows, the average value of the reference group is **m**_0_, and the average value of the comparative group is **m**_1_; the samples of the comparative group are represented by **z**.

$$z={\left[\begin{array}{cccc} E^{1} & E^{2} & \cdots & E^{n} \end{array}\right]}^{T}.$$ (13)

When a normal-state event is *H*_0_ and an abnormal spoofing event is *H*_1_, the probability is defined as Eqs (14) and (15). The event detection using the maximum likelihood method can be performed by Eqs (16) and (17). If the value of an event exceeds 1, it is more likely to be a spoofing event. If the value is less than 1, the event is more likely to be a normal event. Eqs (20) and (21) can be rewritten as Eqs (18) and (19) [18]:

$$p(z|H_{0})=e^{-\frac{1}{2}{\left(z-m_{0}\right)}^{T}R_{n}^{-1}(z-m_{0})},$$ (14)

$$p(z|H_{1})=e^{-\frac{1}{2}{\left(z-m_{1}\right)}^{T}R_{n}^{-1}(z-m_{1})},$$ (15)

$$H_{0}:\Lambda (z)=\frac{p(z|H_{1})}{p(z|H_{0})}=e^{-\frac{1}{2}{\left(z-m_{1}\right)}^{T}R_{n}^{-1}(z-m_{1})}{}^{+\frac{1}{2}{\left(z-m_{0}\right)}^{T}R_{n}^{-1}(z-m_{0})}\leq 1,$$ (16)

$$H_{1}:\Lambda (z)=\frac{p(z|H_{1})}{p(z|H_{0})}=e^{-\frac{1}{2}{\left(z-m_{1}\right)}^{T}R_{n}^{-1}(z-m_{1})}{}^{+\frac{1}{2}{\left(z-m_{0}\right)}^{T}R_{n}^{-1}(z-m_{0})}>1,$$ (17)

$$H_{0}:\text{ln}\;\Lambda (Z)=-\frac{1}{2}\sum_{i=1}^{N}{\left(z_{i}-m_{1}\right)}^{T}R_{n}^{-1}(z_{i}-m_{1})+\frac{1}{2}\sum_{i=1}^{N}{\left(z_{i}-m_{0}\right)}^{T}R_{n}^{-1}(z_{i}-m_{0})\leq 0,$$ (18)

$$H_{1}:\text{ln}\;\Lambda (Z)=-\frac{1}{2}\sum_{i=1}^{N}{\left(z_{i}-m_{1}\right)}^{T}R_{n}^{-1}(z_{i}-m_{1})+\frac{1}{2}\sum_{i=1}^{N}{\left(z_{i}-m_{0}\right)}^{T}R_{n}^{-1}(z_{i}-m_{0})>0,$$ (19)

$$H_{0}:\sum_{i=1}^{N}{\left(z_{i}-m_{0}\right)}^{T}R_{n}^{-1}(z_{i}-m_{0})\leq \sum_{i=1}^{N}{\left(z_{i}-m_{1}\right)}^{T}R_{n}^{-1}(z_{i}-m_{1}),$$ (20)

$$H_{1}:\sum_{i=1}^{N}{\left(z_{i}-m_{0}\right)}^{T}R_{n}^{-1}(z_{i}-m_{0})>\sum_{i=1}^{N}{\left(z_{i}-m_{1}\right)}^{T}R_{n}^{-1}(z_{i}-m_{1}).$$ (21)

In real-time spoofing detection of a GNSS system, a normal-state event and an abnormal-state event cannot be fixed as specific value. This can be addressed by using the sliding window. The samples in the comparative group, **z**, are compared with the average value of those in the reference group. The test statistic can be written as Eq (22) and compared with the threshold used to determine whether a spoofing signal is present. This value is compared with threshold, *T*.

$$DI=\frac{1}{N}\sum_{i=1}^{N}{\left(z_{i}-m_{0}\right)}^{T}\Sigma_{n}^{-1}(z_{i}-m_{0}),$$ (22)

DI>T. (23)

**Fig 3:**
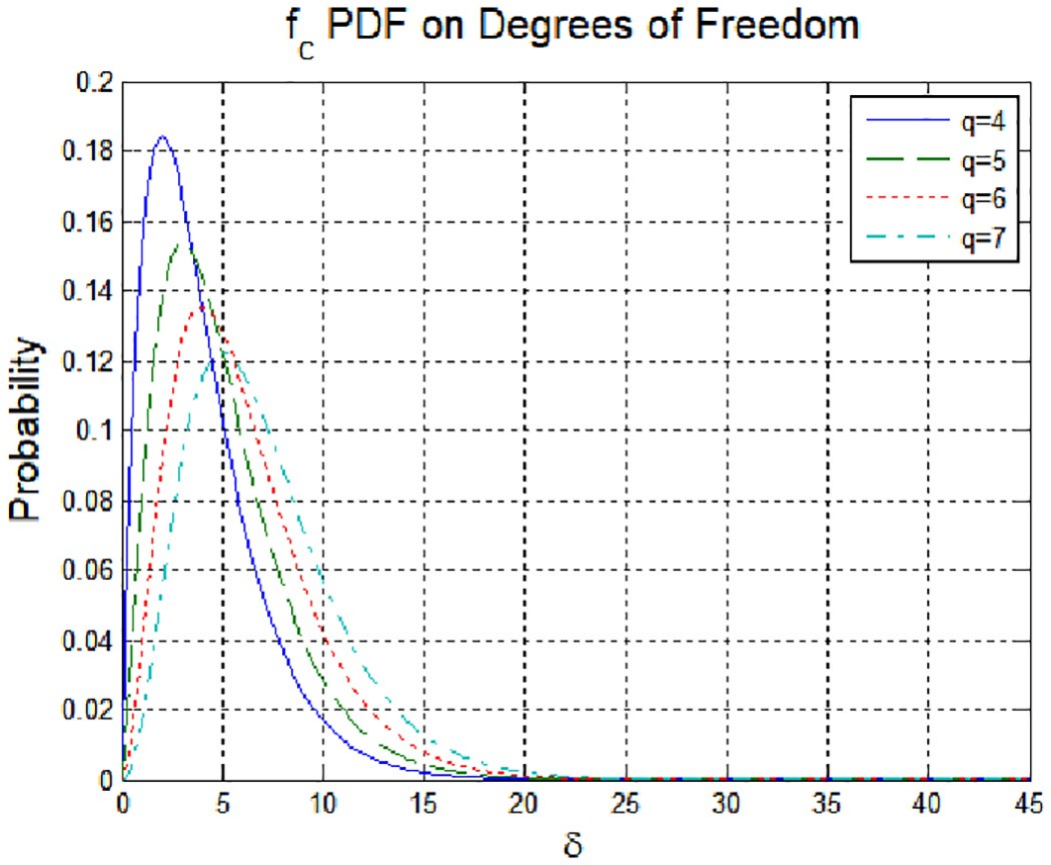
Probability density function according to the degrees of freedom.

If the measured values in the comparative group follow the distribution of those in the reference group and *N* = 1, the probability density function of the test statistic becomes a chi-square distribution, as shown in Fig 3.

**Fig 4:**
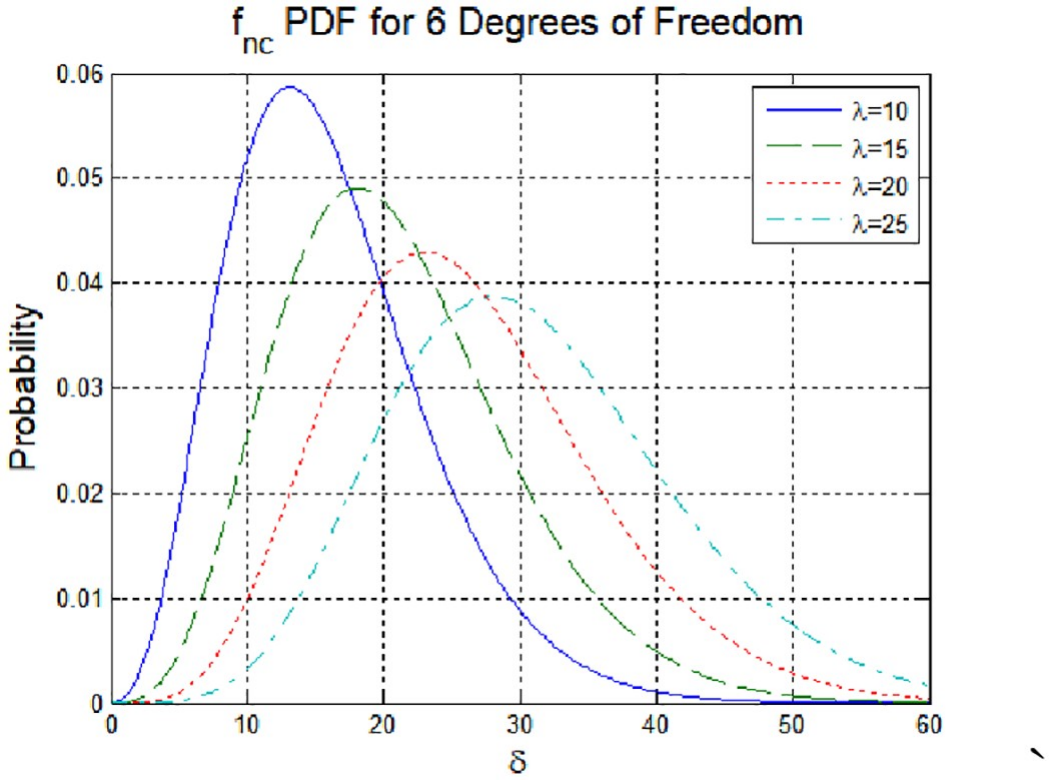
Non-central probability density function (degrees of freedom: 6).

The distribution of the test statistic with changes in the mean value of **z**_*i*_ of the spoofing signal is a non-centered chi-square distribution, as shown in Fig 4.

The variation in the difference between the measured and estimated pseudoranges due to the presence of a spoofing signal is given by Eq (24) [c.f. with Eq (5) [10]]:

$$\begin{array}{ll} \rho_{c}^{i}-\rho_{e}^{i} & =\rho_{s}^{i}+CR^{i}-\rho_{e}^{i}\\ & =\|X^{i}-X_{s}\|+(b+\delta b)+{\tilde{\varepsilon }}_{s}^{i}+CR^{i}-\|X^{k}-X_{u}\|\\ & =\|X^{i}-(X+\delta X_{s})\|+(b+\delta b)+{\tilde{\varepsilon }}_{s}^{i}+CR^{i}-\|X^{k}-(X+\delta X_{u})\| \end{array}.$$ (24)

Here, *ρ*_*s*_ is the pseudorange measured when a spoofing signal is present, *X*_*s*_ is the target position of the spoofing signal, and ${\tilde{\varepsilon }}_{s}$ is the error generated by the spoofing signal. The difference in the pseudoranges is due to variation in the receiver clock error due to the spoofing signal, and to the error generated by the spoofing signal and the difference between the position of the spoofing signal and that of the receiver.

**Fig 5:**
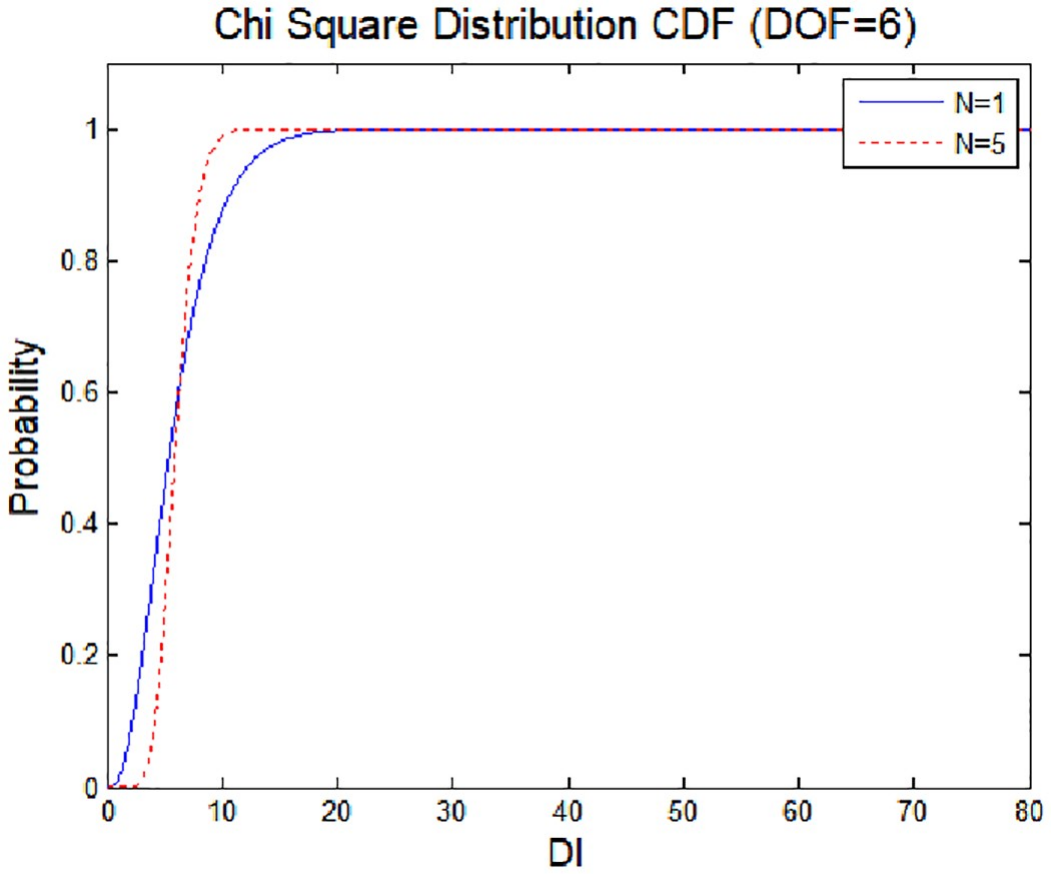
Chi-square distribution (CDF).

Fig 5 shows the cumulative distribution function (CDF) of the chi-square distribution according to the number of comparison groups (*N*); the number of visible satellites is 10 and there are 6 degrees of freedom. The CDF tends to be sloped upward as the number of comparison groups increases. The threshold value for detecting signal abnormalities varies depending on the amount of data accumulated. When the probability of a false alarm is set to *P*_*fa*_ = 10^−7^ in the chi-square distribution according to the normalized values, the false alarm threshold value is 43.35 when the sliding window is not applied (*N* = 1) and is lower, at 17.81, with application of the sliding window (*N* = 5). The effect of application of the sliding window, and thus lowering of the threshold value, can be seen as the data accumulate.

**Fig 6:**
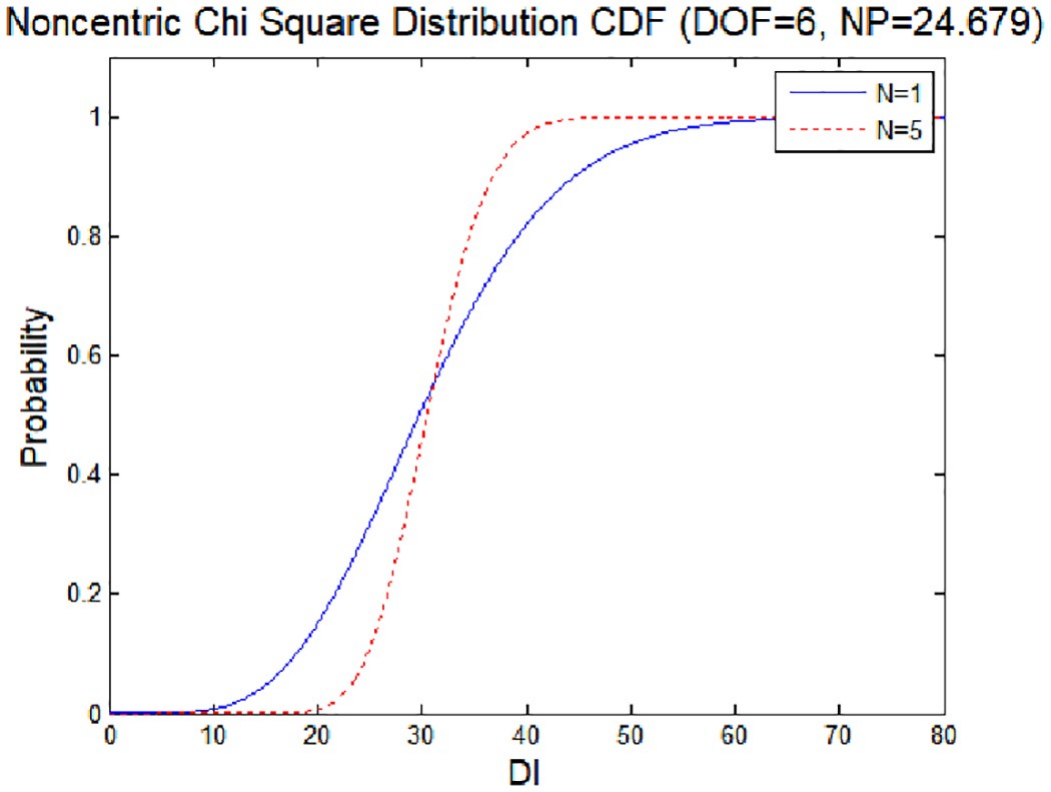
Non-centric Chi-square distribution (CDF).

**Fig 7:**
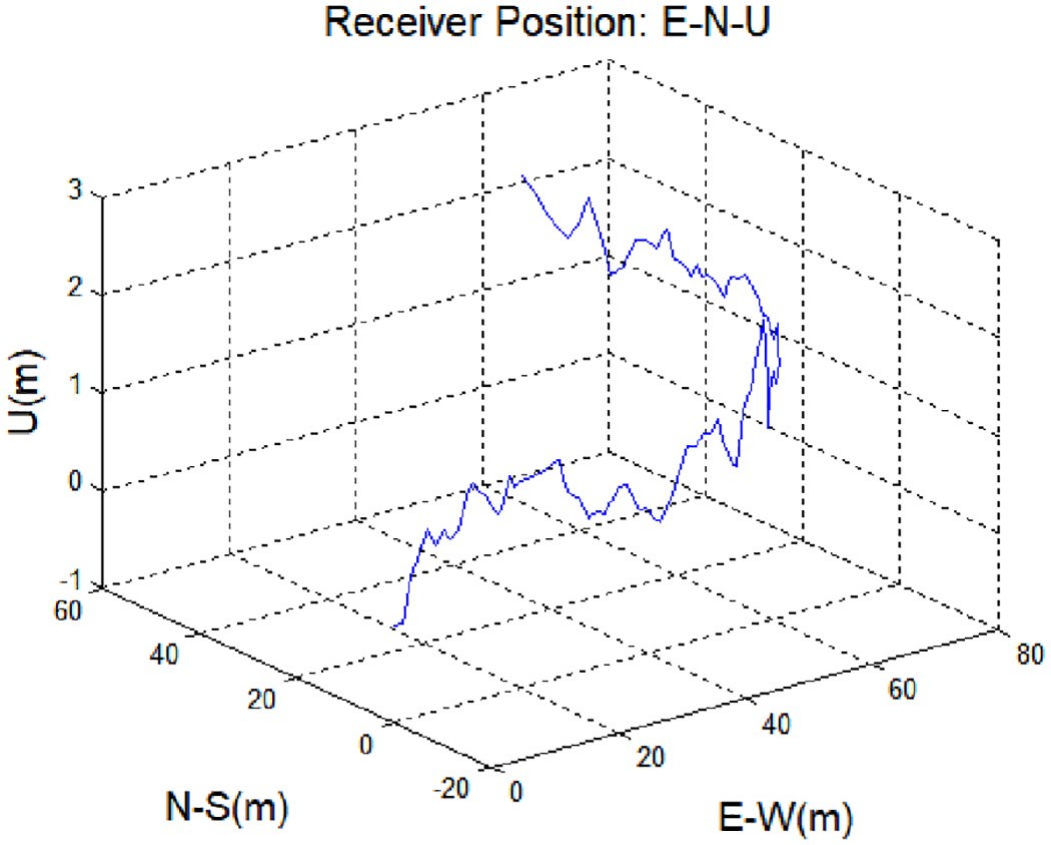
Receiver position during a spoofing attack with all channels subject to a 1.465-m pseudorange delay.

Fig 6 shows the cumulative distribution as the chi-square distribution changes to a non-centric chi-square distribution due to errors. From this graph, the minimum detection error of the detection technique can be derived [19]. The minimum detection error indicates the minimum amount of error data required to detect an anomaly in the signal. Error is given by the probability of an anomaly not being detected, even though an anomaly has in fact occurred. If the probability of miss detection (MD) is set to *P*_*md*_ = 10^−3^ and the threshold value set in Fig 7 is applied, the minimum detection error (MDE) satisfying MD and the false alarm threshold is 24.679. In this case, the detection performance is improved compared with the case where the MDE has a value of 87.470 and is detected using a simple chi-square test. The strength of spoofing (*λ*), is effect to non-centric distribution as Figs 4 and 6. Thus, the spoofing strength influences the threshold value for a false alarm.

Using the method proposed in this paper, it is possible to detect a meaconing attack [(*δb* in Eq (24)] or a spoofing attack aimed at a specific location [*δX*_*s*_ in Eq (24)]; this is not possible with the RAIM method, which checks for a spoofing signal according to the correlations among channels. The difference between the products of Eqs (5) and (18) can be determined using Eq (17). The threshold required to detect a spoofing signal is shown in Fig 5. In addition, it is possible to distinguish a temporary abnormality from a continuous spoofing signal, thus overcoming the limitations of the CUSUM technique, with which determining the steady state average value is difficult. The proposed method relies on an accumulation of real-time measurement values to realize a more robust detection performance.

The proposed method may recognize a signal as a spoofing signal when errors, such as multi-path errors, occur on a continuous basis. However, as mentioned in the Introduction, when the method of detecting a spoofing signal by reference to the receiver’s output, e.g., signal strength, is applied simultaneously, the influence of a spoofing attack and temporary multi-path errors on the signal can be distinguished.

### Simulation results

To improve the accuracy of the navigation solution, the proposed spoofing detection technique was applied to data obtained from a mobile receiver (via a ground-based augmentation system, GBAS) during a simulation. The proposed method detects spoofing signals by monitoring changes in the pseudorange difference before and after a spoofing signal is applied. Although it is possible to detect a spoofing signal without using a GNSS augmentation system, such as GBAS, GNSS augmentation offers improved accuracy before the spoofing signal is applied, and enhances pseudorange differences thereafter. Thus, GNSS augmentation ensures the accuracy of reference group values before a spoofing signal is applied. The data used in the simulation were obtained from an unmanned aerial vehicle (UAV) equipped with a global positioning system (GPS) receiver. The simulation time was 48 s, the data acquisition period was 0.5 s, and the simulated signal was applied 33 s after the simulation began. The UAV starting position was as follows: latitude, 36.39140°; longitude, 127.39769°; and altitude, 111.71 m. This position corresponds to the (0, 0) position in Fig 7(a). During the simulation period, there were 10 visible satellites [pseudo-random noise (PRN) numbers: 23, 3, 28, 12, 17, 6, 2, 19, 9, and 22]. The installed receiver was a NovAtel FlexPak6 system, with a bandwidth of 15 MHz and narrow (0.1) chip correlator spacing. The measurement sampling time was 0.5 s. In the simulation, only the rebroadcasting device was applied based on the receiver’s position. The signal of a meaconing attack is similar to the actual navigation signal; thus, it is not easy to distinguish the spoofing signal. In the simulations, the spoofing signal assumed a pseudorange delay of 1.465 m across all satellite channels.

We acquired simulation data in the real situation while the actual UAV was operated. As a spoofing scenario, pseudorange delay was applied to the data acquired from the receiver. In this scenario, spoofer receives a signal in real time and adds a constant delay to all measurements. When delay was applied to all measurements, the position of the receiver did not change and it is difficult to detect spoofing. As another view point, when spoofing is applied to a part of channels instead of all channels, the rationality between measurement and navigation solution is broken. The spoofing signal can be detected more easily than the current scenario. In addition, spoofing appears differently depending on the conditions. It is also difficult to fix the standard index to analyze detection performance. When a certain delay is applied to all channels, the performance of the algorithm according to the spoofing size can be quantitatively analyzed to select a scenario.

**Fig 8:**
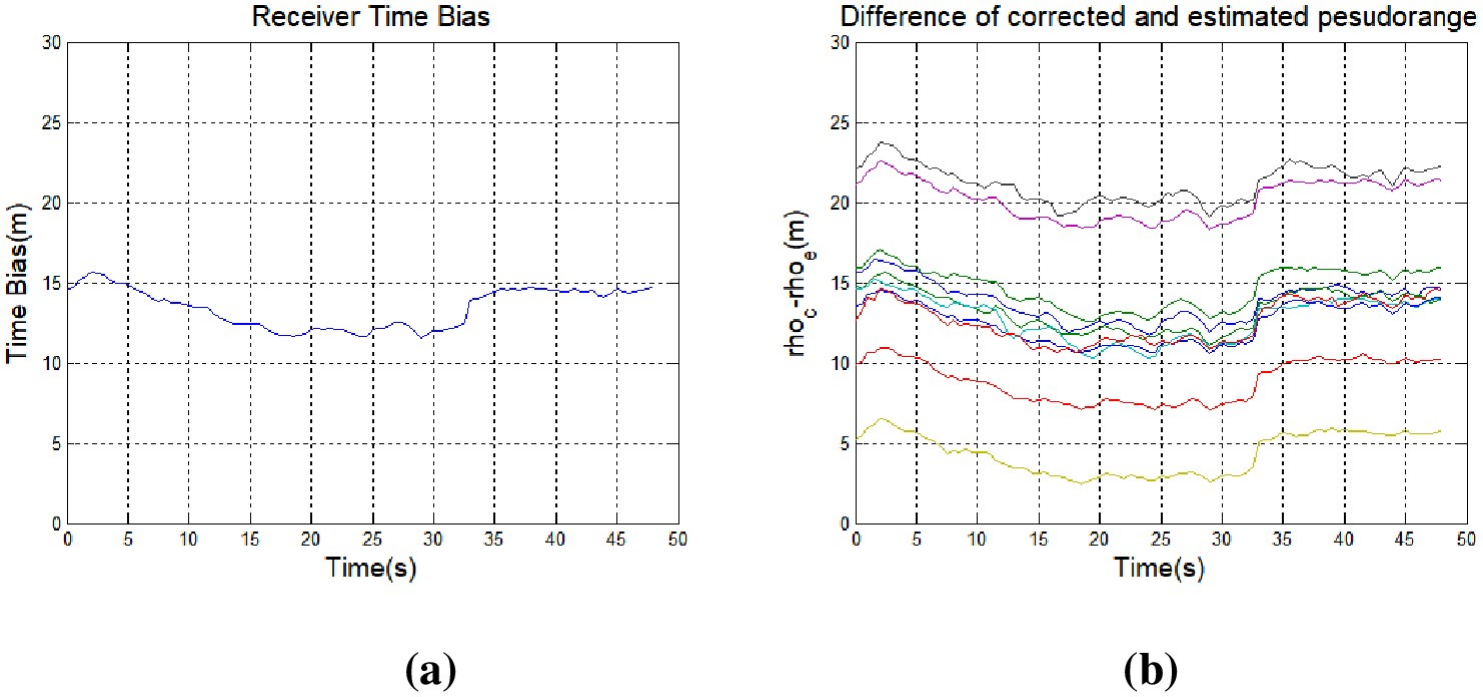
Measurement of receiver position during a spoofing attack with all channels subject to a 1.465-m pseudorange delay: (a) time bias and (b) difference between the corrected and estimated pseudoranges.

Fig 7 shows changes 3-dementional position of the receiver in the presence of a spoofing signal. The same delay was applied to all channels simultaneously, such that the position of the receiver was not affected significantly. It is not easy to detect a spoofing attack based simply on position data. However, as shown in Fig 8(a), the time error with respect to the receiver position changes with input of the spoofing signal. Fig 8(b) shows the difference between the corrected and estimated pseudoranges for each input signal, as well as those corresponding to the steady state and spoofing state, given by Eqs (5) and (24).

**Fig 9:**
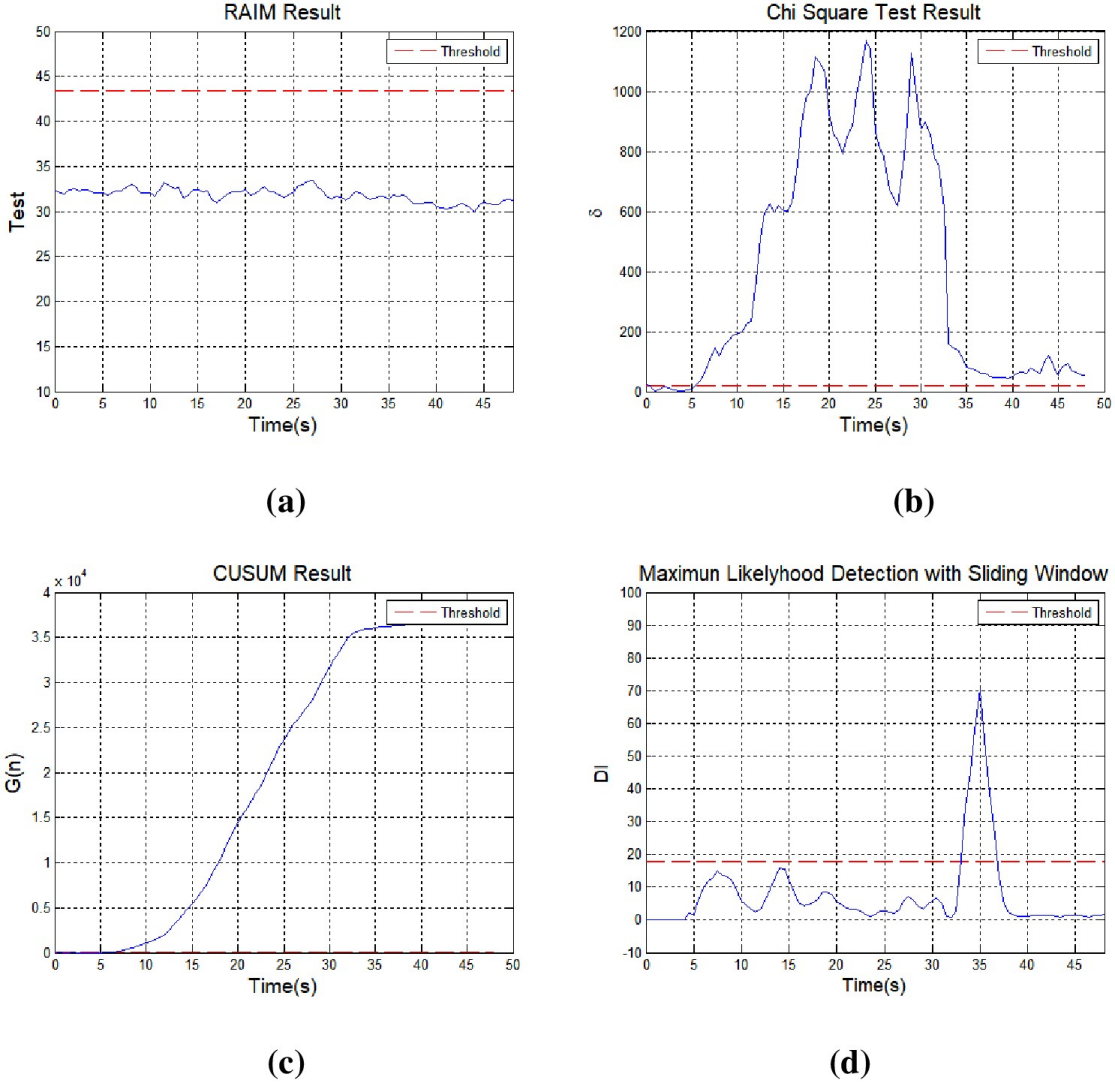
Spoofing signal detection results obtained using receiver autonomous integrity monitoring: (a) RAIM, (b) chi-square test, (c) CUSUM, and (d) sliding window.

The proposed method can detect changes on a continuous basis, even in the case of small positional errors, with spoofing signal input. Fig 9(a) shows the receiver abnormalities detected using the conventional RAIM technique. The false alarm threshold value is 43.35 when the probability of a false alarm is set to *P*_*fa*_ = 10^−7^; there were 10 visible satellites and 6 degrees of freedom. As described above, when using the RAIM method it is not easy to detect a spoofing signal because this method checks for correlations among the measurements [13]. The simulation results show that it is impossible to detect a spoofing signal when a meaconing attack is applied, or the target position based on a reference signal.

Fig 9(b) shows receiver anomalies detected using the chi-square test; with this approach, it is important to set a detection threshold for anomalous signals. Drift from the average initial operating value as the receiver operates is recognized as error. Fig 9(b) shows the test statistics for the chi-square test. A spoofing signal alarm occurred even though no spoofing signal was input.

Fig 9(c) shows the results of spoofing signal detection with application of the CUSUM technique. It can be difficult to determine the presence of an abnormal signal if the mean and standard deviation of the test statistics are not estimated exactly. Similar to the chi-square test, errors gradually accumulated and signal abnormalities occurred (even though the signal was in a normal state) relative to the initial mean and standard deviation. The cumulative sum should be reset once the spoofing signal is terminated in using the CUSUM method.

Fig 9(d) shows spoofing signal detection results when the sliding window method proposed herein was applied. The cumulative sum was set to 5 (*N* = 5), and the test statistic increased after the spoofing signal was applied. A signal exceeding the threshold value for a spoofing signal was detected at 33 s, and the maximum signal was detected at 35 s. Unlike existing techniques, with the CUSUM method, the value of the test statistic changes from the moment of spoofing signal input, allowing for faster detection of the spoofing signal.

**Fig 10:**
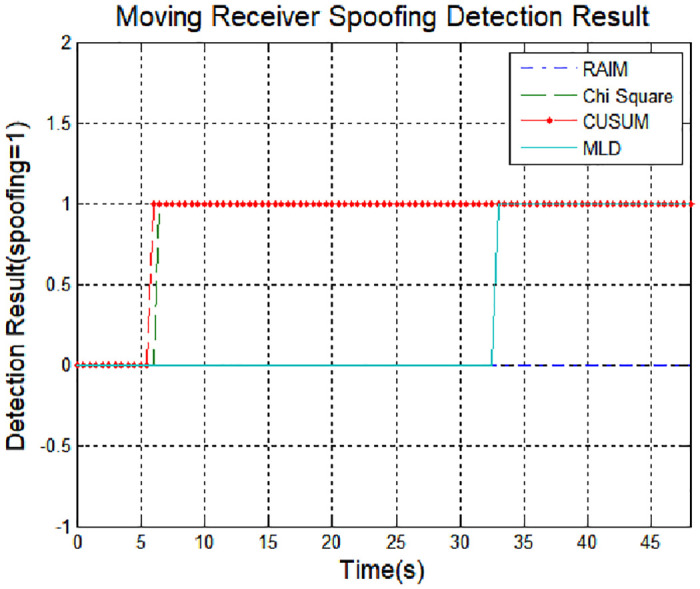
Spoofing detection result.

Fig 10 shows the time to detect spoofing for each algorithm. The RAIM method did not detect spoofing, and the chi-square and CUSUM methods were mistaken for spoofing under normal conditions. In the proposed method, the characteristics of the navigation signal are well reflected and the spoofing signal was normally detected at the time the spoofing signal was applied. Therefore, the proposed method can detect spoofing signal by reflecting changes in the measurement of the receiver during operation time.

**Fig 11:**
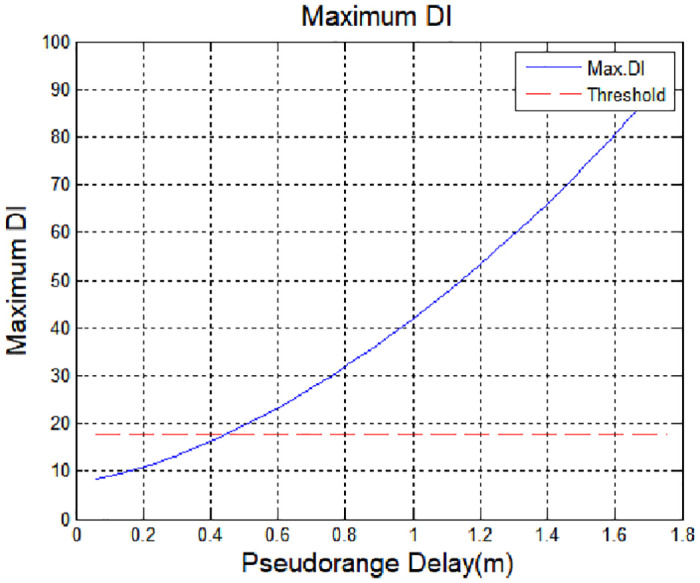
Maximum detection index according to spoofing magnitude.

Fig 11 shows the maximum value of the sliding window technique according to the delay of pseudorange. When this value exceeds the threshold, it is possible to detect spoofing signals. When the spoofing signal was similar to the existing signal, DI was present under the threshold value, so that the spoofing signal could not be detected. The larger the delay value, the easier it was to detect the spoofing signal. In this simulation, it was confirmed that spoofing could be detected when it was 0.47 m delay or more.

**Fig 12:**
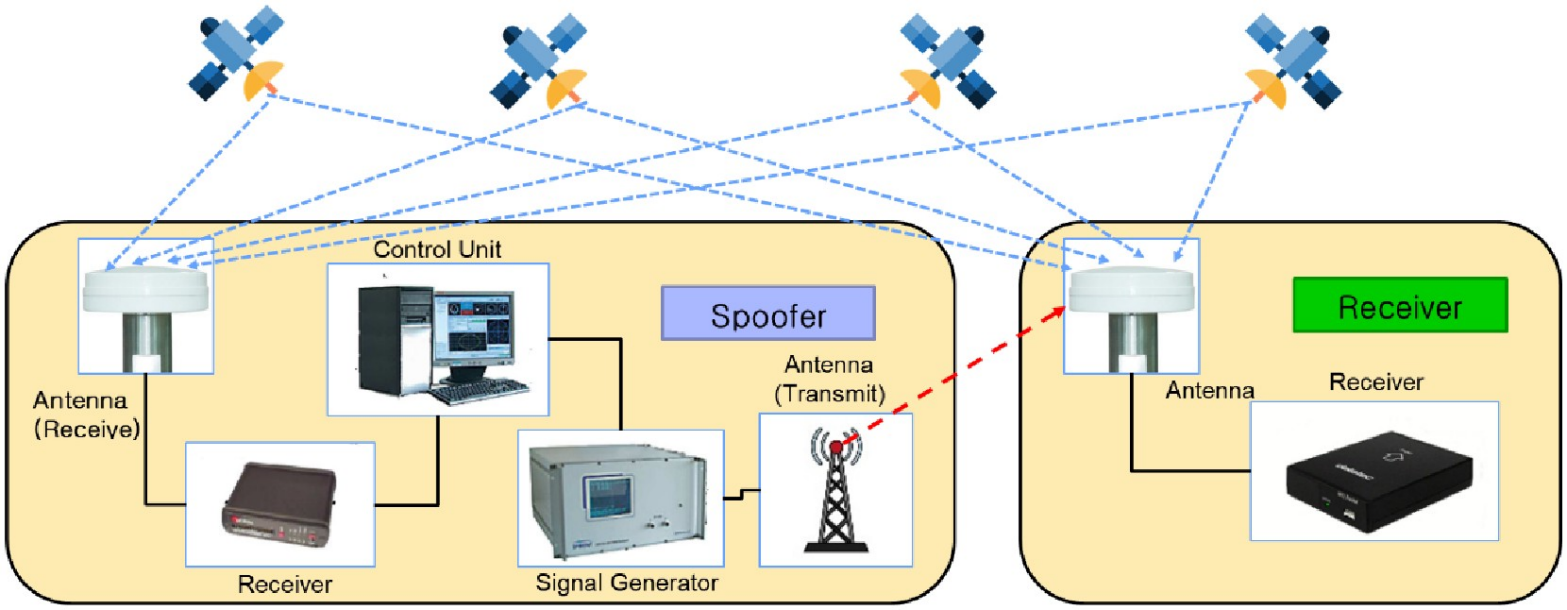
Spoofing signal generator process.

For the evaluation of effectiveness, spoofing detection performance should be analyzed with the characteristic of the spoofing signal generator. Fig 12 is the general shape of the spoofing signal generator. The spoofer usually consists of GNSS antenna, receiver, control computer, signal generator, and transmit antenna. In the case of a spoofer that generates a spoofing signal based on an authorized signal, it has a receiver that receives a GNSS signal for generating a spoofing signal based on the authorized signal. In this process, the signal generator uses the reference clock to generate the signal, and the general reference clock has a sampling rate of 100 ns. Considering the time measurement error ± 20 ns of the receiver, the spoofing detection method could detect a pseudorange error of 80 ns, which corresponds to a distance of 23.44 m. Therefore, the performance of proposed algorithm could be an effective discrimination algorithm considering the general spoofing generation [20].

**Fig 13:**
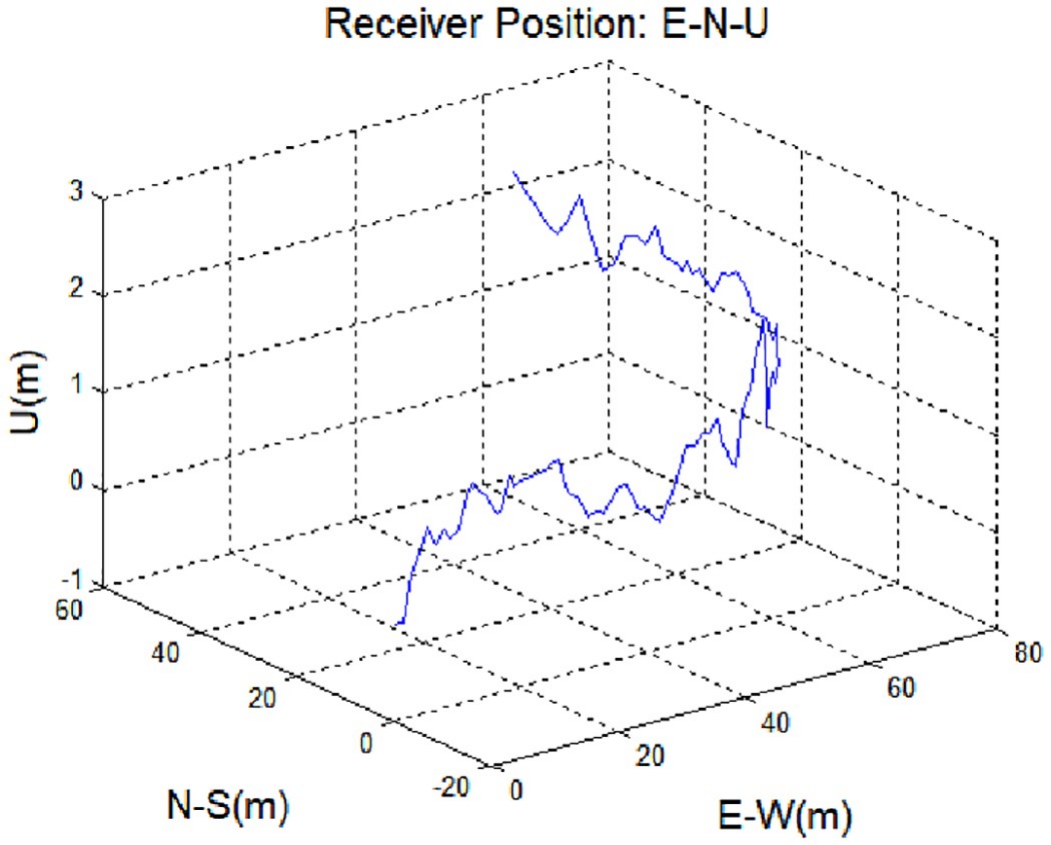
Receiver position during application of a spoofing signal to a single channel, with a 5.86 m delay.

**Fig 14:**
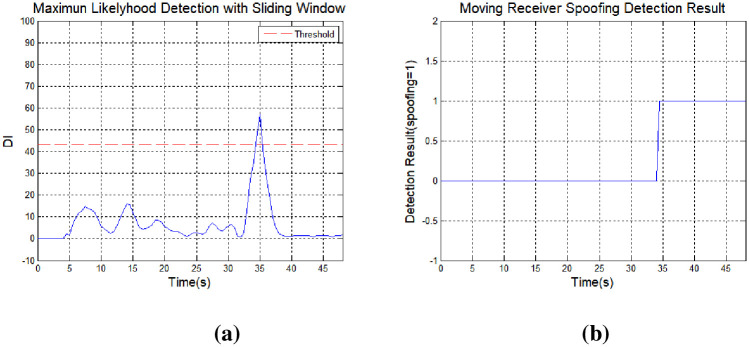
Spoofing signal detection results obtained using the sliding window method (single-channel spoofing): (a) DI and (b) detection results.

The effect of applying a spoofing signal to a single channel (PRN 23), subject to a 5.86-m delay, was determined. The receiver position was changed by the spoofing signal (Fig 13), unlike the case when a spoofing signal is applied to all channels. Fig 14 shows the detection results with the spoofing signal applied to a single channel. It can be seen that the delay is greater versus the case where the spoofing signal is applied to all channels. Because spoofing detection is based on the values obtained at all channels, there is a delay in single-channel spoofing detection. The spoofing signal detection performance can be determined based on the analysis shown in Fig 5.

As shown by the simulation results, the proposed method monitors pre- and post-spoofing changes in the signal relative to the measured values by applying the sliding window, and is able to detect spoofing signals that cannot be distinguished by existing methods. Also, it prevents false alarms, thereby improving spoofing signal detection performance.

## Conclusion

To improve the ability to detect spoofing attacks on satellite navigation systems, the detection index and detection method should be set according to the characteristics of the navigation signal. An attacker can disrupt the navigation signal using various methods. Therefore, it is desirable to check for spoofing signals using a multifaceted approach. The method proposed in this paper is applicable to both fixed and mobile receivers, as well as to various types of spoofing attacks, as it checks for signal anomalies based on measurements of the navigation receiver, without the need for any special hardware. By applying the sliding window method, the navigation data collected in real time can be applied for detection of spoofing attacks. The accumulation of data over a certain period of time improves the reliability of spoofing signal detection; additionally, detection errors that occur when using the existing methods can be reduced by comparing the signal before versus after spoofing. The proposed method is based on comparison of the characteristics of steady-state navigation signals and spoofing signals. Because it continuously monitors the signal, it can be applied for detection of various types of spoofing attacks, including meaconing attacks; additionally, the method could be used in future to improve the reliability of receiver position measurements. However, since spoofing signals vary according to the type of spoofing attack, it is desirable to apply several methods simultaneously to detect spoofing signals. We plan to continue optimizing the proposed maximum likelihood-based sliding window method in future work, to further enhance detection performance.

## Supporting information

S1 File Flight 1. (MAT)
